# Application of MEMS Sensors for Evaluation of the Dynamics for Cargo Securing on Road Vehicles

**DOI:** 10.3390/s21082881

**Published:** 2021-04-20

**Authors:** Jozef Gnap, Juraj Jagelčák, Peter Marienka, Marcel Frančák, Mariusz Kostrzewski

**Affiliations:** 1 Department of Road and Urban Transport, Faculty of Operation and Economics of Transport and Communications, University of Zilina, 010 26 Zilina, Slovakia; juraj.jagelcak@fpedas.uniza.sk (J.J.); peter.marienka@fpedas.uniza.sk (P.M.); marcel.francak@fpedas.uniza.sk (M.F.); 2 Department of Fundamentals in Means of Transport, Faculty of Transport, Warsaw University of Technology, 00-662 Warsaw, Poland; mariusz.kostrzewski@pw.edu.pl

**Keywords:** dynamic events, acceleration, MEMS, mean fully developed deceleration, transport, cargo securing

## Abstract

Safety is one of the key aspects of the successful transport of cargo. In the case of road transport, the dynamics of a vehicle during normal events such as braking, steering, and evasive maneuver are variable in different places in the vehicle. Several manufacturers provide different dataloggers with acceleration sensors, but the results are not comparable due to different sensor parameters, measurement ranges, sampling frequencies, data filtration, and evaluation of different periods of acceleration. The position of the sensor in the loading area is also important. The accelerations are not the same at all points in the vehicle. The article deals with the measurement of these dynamic events with MEMS sensors on selected points of a vehicle loaded with cargo and with changes in dynamics after certain events that could occur during regular road transport of cargo to analyze the possibilities for monitoring accelerations and the related forces acting on the cargo during transport. The article uses evaluation times of 80, 300, and 1000 ms for accelerations. With the measured values, it is possible to determine the places with a higher risk of cargo damage and not only to adjust the packaging and securing of the cargo, but also to modify the transport routes. Concerning the purposes of securing the cargo in relation to EN 12195-1 and the minimum values of forces for securing the cargo, we focused primarily on the places where the acceleration of 0.5 g was exceeded when analyzing the monitored route. There were 32 of these points in total, all of which were measured by a sensor located at the rear of the semi-trailer. In 31 cases, the limit of 0.5 g was exceeded for an 80-ms evaluation time, and in one case, the value of 0.51 g was reached in the transverse direction for a 300-ms evaluation time.

## 1. Introduction

Every day, accidents and damage happen in traffic on European roads as a result of the incorrect positioning or securing of cargo on trucks. The European Agency for Safety and Health at Work estimated that up to 25% of truck accidents are caused by an insufficient securing of cargo. Therefore, it is crucial to prevent cargo from sliding, turning over, moving, deforming, or rotating in any direction during transport in order to protect the health and life of people as well as to protect the assets. The fundamental prerequisite for safe cargo transport is as follows: the sum of forces acting in the direction of each axis must equal zero, and similarly, the sum of moments must be equal to zero. To identify the forces acting on a vehicle and the cargo, it is important to measure them using appropriate devices equipped with an accelerometer and a gyroscope. These are the reasons which make the application of microelectromechanical system (MEMS) sensors appropriate.

The main research question of the article is to recognize whether it is possible to apply the available MEMS sensors for monitoring the accelerations acting on the cargo in road transport during braking tests or regular transport of cargo by various vehicle combinations. In the article, we will answer how the measured data can be used in the context of increasing the safety of transport services.

MEMS sensors have been used in various technical solutions and in analyses of state of road and state of vehicle for last two decades. The research connected to MEMS sensors takes into account the dynamics of driving, the quality affecting ride comfort, driving safety, fuel consumption, and other factors of road transport safety. It is worth mentioning that such research can be analyzed both for roadways (pavement) and for road vehicles—in most cases, separately. The literature review, presented in the following section, allowed to abstract numerous studies connected separately to applications of MEMS sensors in roadway measurements (presented in the first paragraph of the next section) and in road vehicle measurements (presented after). The literature review allowed to determine a research gap as well - this research gap is mentioned in the end of the next section of this paper.

## 2. Literature Review

The authors of [1] developed and described the application of a system for road users and operators to monitor pavement roughness. Their vibration-based system consisted of a low-cost three-axis MEMS accelerometer and a global positioning system (GPS) sensor (this is a typical combination of devices for the described solutions), which, together with an Arduino board, were embedded in a road vehicle in order to monitor roads’ condition and potential anomalies. The authors proved that such a solution is less expensive than previously known as [2,3,4]. It was also less expensive than at least one another device using the Arduino board presented in [5]. According to [6], a system with the usage of a MEMS can indicate in the following road abnormalities: potholes and bumps, cracks, etc. Such abnormalities affect safety and the comfort of riding and lead to a reduction in a vehicle’s lifecycle [7]. The possibility to detect the softness of gravel roads with MEMS sensors installed onboard of a passenger car was studied in [8]. Apart from the mentioned aspects, the state of safety is also affected by driving style. Therefore, the MEMS-based system presented in [9] consisted of not only an accelerometer but also a magnetometer and a gyroscope. Such a system allows to monitor a road vehicle’s movement parameters and, above all, driving style in order to exclude certain dangerous situations on roads [9]. The approach, which enables accurate estimation of the road uphill gradient for both moving and parked vehicles was developed in [10]. An analysis of a system applied in order to determine whether a vehicle is on an overhead road was presented in [11]. The authors of [12] claimed that road defects assessment was one of the most important problems that could be dealt with by utilizing numerous accelerometers mounted on vehicles. Therefore, they proposed such a system using a MEMS and drivers’ smartphones.

Apart from road infrastructure analyses, MEMS-based solutions are applied to research the mutual influence on both roads and vehicles as in [13,14]. The authors of [13] measured vehicle-road interaction with a high sampling rate and better position accuracy, whereas the authors of [14] developed MEMS-based system in order to assess the condition of the pavement, the condition of the vehicle, and the passengers’ comfort.

As far as traffic is concerned, it is at the center of interest of various research teams. In [15], the presence of vehicles and their travel direction and speed were determined in order to ensure traffic safety and support the concept of intelligent streets. The concepts presented in [16,17] were similar in their aim. Both research teams claimed that having knowledge of the exact position and velocity of each car and a driver assistance system can allow to find the fastest route and to provide reliable warnings to prevent potential accidents at the end of traffic jams. The authors of [17] focused on improvement of road traffic efficiency using optimal traffic distribution. In [18], a system dedicated to the detection, classification, and determination of the direction of vehicles traveling on two-lane roads was proposed.

MEMS are also used in research from other perspectives. In [19], the authors used a MEMS-based system in order to deal with certain abnormalities in driving state directly connected to human behaviors as detection of loss of vigilance, perilous driving, drowsiness, sleep deprivation and any mobile phone usage during driving.

According to the authors of [20], application of MEMS accelerometers can achieve a higher throughput of data. Nevertheless, up-to date, the authors used simulation models in order to study an accident impact analysis to minimize occurrence of incidents.

A slightly different system was developed in [21]. This system detects the occurrence of a road accident, informs the victim’s family, connects the nearest hospital’s staff to inform them about the incident. Additionally, the authors of [22] researched an automatic vehicle tracker system, which was developed to remove some delays of accident incidence happened on roads network. Another group of researchers developed an automatic early warning system for accidents and providing location data with a MEMS in order to decrease the number of deaths on roads caused by a lack of information for emergency medical respondents and police officers [23].

A significant subject of research in various systems using MEMS sensors is measurement errors. In [24], the authors mentioned that improvement of such systems performance is necessary, especially for precise data on velocity derived from GPS. The authors of [25] recognized that measurements of systems with use of MEMS are affected by issues such as noise, biases due to the lever arm between wheels and sensors in on-board road vehicle systems, inertial measurement unit (IMU) cross-axis effects, etc. Use of gyroscope observables to complement the accelerometer may exclude these flaws [25].

The authors of [26] explored the idea of using the smartphone to conduct GPS and inertial navigation system integration for car navigation (the traditional technique for this integration is based on Kalman filtering with a dedicated inertial sensor module consisting of three orthogonal gyroscopes and three orthogonal accelerometers [27]).

In [28], an IMU was additionally equipped with MEMS and GNSS receivers in aided inertial navigation systems for automatic driving vehicles. The authors of [29] developed a system with a MEMS for alternate lane determination, which can be used either for high-occupancy vehicles or autonomous vehicles. The standard positioning service GPS, the MEMS IMU, GNSS, and commercially available road-level network maps are used in the researchers’ system. The authors claimed that real road experiments showed a 97.14% success rate of the system [29]. A GPS receiver and navigation system were integrated with two horizontal low-cost MEMS-grade accelerometers and a single vertical MEMS gyroscope, and a vehicle odometer as a whole system was also earlier analyzed in [30]. The authors obtained around a 40% improvement in the positional error of GPS.

According to the [31], measurements by the MEMS would degrade quickly during the frequent discontinuity of signals in urban environments, and in order to exclude potential error growth, two methods were analyzed, namely (1) improving the accuracy of the MEMS IMU stochastic model by Allan variance analysis [32] and then initialization of Kalman filtering for sensor parameters, and (2) including a body velocity constraint and improved zero velocity updates [31,32]. Other researchers have worked on error elimination as well. In [33] distributed dual-H∞ filtering was applied to reduce the impact of the uncertain nonlinear drift effect of MEMS inertial sensors.

The authors of [34], to exclude any indifferences when GNSS is used, proposed a method to decrease the influence of vehicles’ position information by fixing it when zero velocity was detected [34]. The authors described their new proposal in [35]. Meanwhile, in [36], the authors addressed the problem of positioning error growth due to gravity leakage by proposing an error containment mechanism. The authors of [37] excluded the nonlinear error characteristics in the integrated navigation system by introducing the concept of mixture particle filtering (MPF). That same year, the researchers explored the benefits of using parallel cascade identification, through which they found a nonlinear system identification technique that improves the overall navigation solution by modeling residual pseudorange correlated errors to be used with Kalman filtering [38].

The authors of [39] also worked on error contribution in a reduced inertial sensor system. They proved that map matching significantly improves the system’s efficiency.

In [27,40,41,42], the researchers developed MPF for their reduced inertial sensor system. This filtering was introduced due to the inherent errors of MEMS inertial sensors and their large stochastic drifts. This solution can be used for lower number of satellites.

In [43], the authors discussed a solution that supports the prediction and compensation of linear and nonlinear inertial errors, which leads to enhanced accuracy of the MEMS during GPS outages.

In [44], the authors used means of a photogrammetric approach in order to improve the positioning by exploiting the overlap between adjacent images. A year later, the authors published the results of system analyses with four instead of three GNSS receivers applied in the system [45].

In [46], the researchers mentioned that MEMS-based systems are inherently immune to signal jamming, spoofing, and blockage vulnerabilities; nevertheless, GPS influences the performance of MEMS-based systems, which evokes error characteristics of a stochastic nature. To deal with such errors, the autoregressive processes for raw data or the correction of GPS positioning during outages by using nonlinear modeling of the inertial navigation system position accompanied by neuro-fuzzy modules can be used.

The authors of [47] recognized that imprecisions of MEMS-based navigation sensors resulted in a high noise level and large, stochastic bias instabilities. They suggested to use a neuro-fuzzy module in order to provide a reliable prediction of the vehicle position during GPS outages.

Most of the abovementioned research was conducted for passenger vehicles. Meanwhile, in [48], the authors researched a system with a MEMS in an agriculture vehicle for collecting information needed to keep the relative position from the impact of narrow, uneven, and complicated conditions. The authors of three consecutive publications [49,50,51] elaborated an integrated positioning MEMS-based system for autonomous off-highway vehicle use (the system was tested on an agricultural utility vehicle). In [52], the author described research on active boom noise damping strategies implemented on a large sport utility vehicle.

The authors of [53] verified their system with the use of a MEMS on signals recorded in both trucks and passenger cars in actual conditions of everyday driving tasks. Analyses of heavy road vehicles were taken into consideration in [54] and [55]. The authors of [56] analyzed an all-terrain vehicle with three-axis MEMS accelerometers installed on various parts of the vehicle’s surface.

The concept of smart tires, with use of MEMS, was taken into consideration in [57,58,59]. The authors of [60] analyzed a real-time estimation of tire/road friction coefficients based on sensor measurement on the basis of the vehicle’s lateral dynamics.

Braking tests with truck were performed in [61] with MEMS sensors. Whereas the paper [62] deals with issues of braking in a passenger car.

The authors of [63,64] dealt with the cargo distribution and its impact on mean fully developed deceleration, braking distance, and braking time on light commercial vehicles.

The above-described brief literature review can be summarized by indication of general topics considered in the research connected to MEMS sensors used in road transport. These topics are as follows:

- Measurements of actual roadways’ state [1,2,3,4,5,6,7,8,12,14];
- Mutual influence analyses on both roads and vehicles [13,14];
- Traffic safety, support, and distribution [15,16,17,18];
- Automatic early warning systems in the case of accident incidence on a road system [7,17,19,21,22,23];
- Measurements of actual vehicle state [9,10,11,12,13,18,25,29,30,31,34,35,42,43,53,54,55,61,62,63,64];
- Correction of positioning errors and other measuring inadequacy in road transport MEMS-based applications [11,24,25,30,31,32,33,36,37,38,39,43,46,47,49,50,51];
- Various aspects in the use of vehicles other than passenger cars (such as heavy road vehicles, trucks, agricultural vehicles, so-called all-terrain vehicles, and even sports cars) [48,49,50,51,52,54,55];
- Smart tires’ measurements [57,58,59,60];
- Indication of significant potential accelerations for cargo damage [61].

As can be observed based on the literature review and the above presented summary, the following research gap is observed. Whereas passenger vehicles are quite often an interest of measurement with MEMS sensors, cargo vehicles face the opposite situation. Only two references connected to analysis of MEMS-based measurements for cargo vehicles were found in acclaimed scientific databases (Scopus, Web of Science) in the analyzed period of time, namely [52,61]. Safety in cargo transportation, both from the viewpoint of the whole traffic and the vehicle carrying the load, is one of the key aspects of the successful realization of road transport processes. The dynamics of such a vehicle during regular events such as braking, steering, and evasive maneuvers are variable in different places of the vehicle, and in the same way, it is one of the transport’s safety factors. The mentioned dynamics can be measured with support of MEMS sensors. Based on the conducted literature review, it can be stated that there is lack of publications that present analyses of safe cargo transport in adequate vehicles. Consequently, the authors of the current paper supposed this to be a research gap, namely the measurement of cargo vehicles’ state with application of MEMS solution. Therefore, the focus of the current paper is on MEMS sensor application developed for freight transport.

## 3. Materials and Methods

According to [65], microelectromechanical systems (MEMS) were first applied in engineering science in the 1960s. Since then, they have developed into a physical–mathematical multi-discipline. The authors of [65] presented a comprehensive literature review and description of MEMS technology. Additionally, the research presented in the paper [66] was elaborated, with particular interest in denoising the dynamic MEMS gyroscope measurements in practical applications. Since the current paper’s aim is to present the application of the MEMS concept, the reader is politely asked to refer to the technical aspects of MEMS technology in external references such as [65,66]. Nevertheless, the authors present the details of the MEMS applied in the current research.

### 3.1. MEMS Sensors Used to Measure Dynamic Actions

For the purpose of measuring dynamic actions during braking with a vehicle and during transport, we used three kinds of MEMS sensors, which were supplemented with a device for measuring the dynamics acting on the vehicle; this device measured and calculated some additional indicators needed for the evaluation of dynamic actions. The combinations of MEMS sensors applied in this research are presented in next subsections, namely: XL Meter; BOSCH BMI260 IMU + UBlox UBX-M8030-CT; BOSCH BHA250 + BOSCH BMG250 + UBlox UBX-M8030-CT; BOSCH BMI160 + UBlox UBX-M8030-CT. The selected parameters of the MEMS sensors are presented in Table 1.

#### 3.1.1. XL Meter

XL Meter (noted further as sensor A) is a battery-operated universal acceleration/deceleration meter with an alphanumeric liquid crystal display (LCD), built-in service brake performance or acceleration evaluation program, and a PC interface. XL Meter uses a complete acceleration measurement system. It consists of a sensor and signal conditioning circuitry, which implements a force–balance control loop. XL Meter is capable of measuring both positive and negative values to a maximum level of ±2 g. The characteristic of the acceleration sensor assures the quality of measurement results. XL Meter uses a differential capacitive acceleration sensor, and it is designed to measure acceleration in two axes (longitudinal and lateral) [67].

Besides the maximum acceleration values in given directions, sensor A measures other indicators:

- *s_0_*—braking distance in meters, which was calculated by the duplicated integration of the acceleration data in the interval of the brake time;
- *v_0_*—initial braking velocity in km/h, which was calculated by the simple numerical integration of the acceleration data in the interval of the brake time;
- *T_br_*—total braking time in seconds, which was calculated by subtracting the brake start time from the brake end time;
- *d_m_*—mean fully developed deceleration (MFDD), which was calculated as follows [67,68]:

(1) $$d_{m}=\frac{v_{b}^{2}-v_{e}^{2}}{25.92\;\left(s_{e}-s_{b}\right)}$$

where *v*_0_—initial velocity of the vehicle given in km/h; *v_b_*—velocity of the vehicle at 0.8 *v_o_* given in km/h; *v_e_*—velocity of the vehicle at 0.1 *v_o_* given in km/h; *s_d_*—distance driven by the vehicle between *v_o_* and *v_b_* given in meters and *s_e_*—distance driven by the vehicle between *v_o_* and *v_e_* given in meters [68].

#### 3.1.2. BOSCH BMI260 IMU + UBlox UBX-M8030-CT

This device (noted further as sensor B) is a combination of an IMU unit and a GPS module. The BMI260 (BOSCH, Gerlingen, Germany) is an ultra-low power IMU that combines precise acceleration and angular rate measurements with intelligent on-chip motion-triggered interrupt features. The 6-axis sensor combines a 16-bit triaxial gyroscope and a 16-bit triaxial accelerometer in a compact Land Grid Array (LGA) package. The IMU provides highly accurate step counting and motion detection. BMI260 has a self-calibrating gyroscope using motionless Component Retrimming (CRT) functionality to compensate for typical MEMS soldering drifts. The IMU includes hardware and time sync functions and also supports a low-latency secondary interface with accelerometer and gyroscope data output [69].

The UBX-M8030 high-performance standard precision GNSS chip (Ublox, Thalwil, Switzerland) provides exceptional sensitivity and acquisition times for all GNSS systems. The chip utilizes concurrent reception of up to three GNSS systems (GPS/Galileo together with either Beidou or GLONASS). Reception from more than one constellation simultaneously allows extraordinary positioning accuracy in urban canyons, even with weak signals and high dynamics. UBX-M8030 also supports message integrity protection, geofencing, and spoofing detection with configurable interface settings. The chip is used for portable applications with demanding size and cost constraints, including rigorous automotive quality and manufacturing standards, extended testing, and low failure rate [70].

#### 3.1.3. BOSCH BHA250 + BOSCH BMG250 + UBlox UBX-M8030-CT

The devices marked further as sensors C and D consist of three MEMS sensors—a combination of an accelerometer, a gyroscope, and a GPS module. The BOSCH BHA250 is a small, low-power smart-hub with an integrated three-axis accelerometer enriched with a programmable microcontroller, all specifically designed to enable always-on motion sensing. Furthermore, it contains software and algorithms for motion-step, gesture, and activity recognition. The overall concept perfectly matches the requirements of smartphones, wearables, or any other application which demands highly accurate, real-time motion data at very low power consumption. The device integrates a millionfold proven 14-bit acceleration sensor. BOSCH BHA250 is targeted for applications such as activity recognition of standing, walking, running, biking, or in vehicle; human-machine interface (HMI) interfaces including gesture detection of motion, tilt, pickup, wake up, and glance; step detection and step counting; indoor navigation and Pedestrian Dead Reckoning (PDR); augmented reality, immersive gaming; tilt-compensated eCompass, and orientation [71].

The BOSCH BMG250 is a three-axial gyroscope consisting of a state-of-the-art low-power 3-axis gyroscope. It has been designed for low-power, high-precision multi-axis applications. BMG250 in the LGA package has a typical power consumption of 850 µA, enabling always-on applications in battery-driven devices. The gyroscope provides high-precision sensor data together with accurate timing of the corresponding data [72]. The UBlox UBX-M8030-CT GPS module was described in the previous subsection.

#### 3.1.4. BOSCH BMI160 + UBlox UBX-M8030-CT

The BOSCH BMI160 (noted further as sensors E and F) is an inertial measurement unit consisting of a state-of-the-art 3-axis, low-g accelerometer and a low-power 3-axis gyroscope. It has been designed for low-power, high-precision 6-axis and 9-axis applications in multiple devices. Due to its built-in hardware synchronization of the inertial sensor data and its ability to synchronize data of external devices such as geomagnetic sensors, the BMI160 unit is ideally suited for augmented reality, gaming, and navigation applications, which require highly accurate sensor data fusion. The BMI160 unit provides high-precision sensor data together with accurate timing of the corresponding data [73]. The UBlox UBX-M8030-CT GPS module was defined in the previous subsection.

### 3.2. A Theoretical Course of Braking and Forces to Design the Securing of Cargo

Cargo vehicles, especially tested semi-trailer vehicle combinations with a gross mass of up to 40 tonnes, are vehicle combinations consisting of two vehicles: a tractor and a semi-trailer. Cargo vehicles behave differently because the main factors influencing vehicle dynamics are the cargo itself, its mass, and the center of gravity. Individual cargo has different dynamics. Cargo vehicles have less braking deceleration and stability than passenger vehicles and a lower traveling speed on the roads. To study the dynamics of a semi-trailer vehicle combination and cargo, more sensors are required because different accelerations can occur in different places of a semi-trailer as well as of cargo. Passenger vehicles are broadly available for tests, but it is difficult to obtain different vehicle combinations for extensive tests with real cargo where damage to cargo and the packaging can occur.

The value of braking deceleration during vehicle braking is not constant. Braking, or stopping of the vehicle, can be divided into the following phases:

- Driver’s response time *t_r_*—the time of the driver’s reaction is defined as a time period which elapses from the moment when the driver accepts an impulse to start braking until the moment when they touch the brake control;
- Brake response time *t_0_*—the time from the moment when the driver starts acting on the brake control until the moment when the pressure in the braking system starts increasing, i.e., until the moment when the vehicle starts braking;
- Braking effect start-up time *t_n_*—the time from the moment when the driver starts acting on the braking control until the moment when the pressure achieves 75% of the nominal pressure of the braking system in the most distant brake cylinder of the braking system; thus, the braking effect start-up time also includes the brake response time;
- Effective braking time *t_ub_*—the time from the end of the braking effect start-up time until the vehicle stops;
- Total braking time *t_cb_*—the time from the moment when the driver touches the brake control until the moment when the car stops; thus, the total braking time is a sum of the braking effect start-up time and the effective braking time;
- Time to stop *t_z_*—the time from the acceptance of an impulse to start braking by the driver until the moment when the car stops; thus, it is a sum of the response time, the braking effect start-up time, and the effective braking time [74].

**Figure 1:**
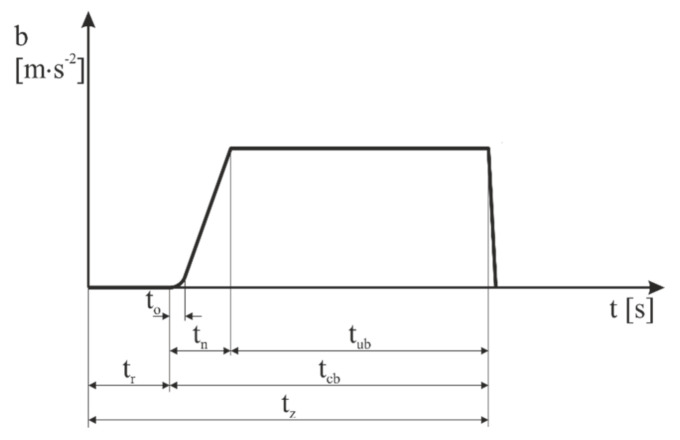
A theoretical course of braking [74].

The theoretical course of the braking deceleration is represented in Figure 1.

According to the standard EN 12195-1:2010 [75] and the CTU Code [76] (CTU = cargo transport units), the following terms are used in relation with the inertial forces considered for the design of cargo securing (Table 2):

- *c_x_*—longitudinal acceleration coefficient;
- *c_y_*—transverse acceleration coefficient;
- *c_z_*—coefficient of acceleration vertically down.

**Figure 2:**
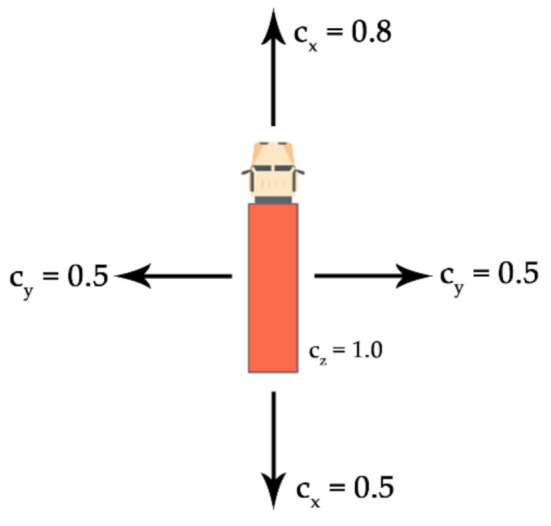
Illustration of applicable acceleration coefficients for road transport [75,76].

For example, an acceleration coefficient of 0.8 in a given direction means that it is necessary to secure 80% of the weight of the cargo in a given direction. To prevent cargo from moving, the cargo has to be secured in longitudinal and transverse directions according to the worst combination of horizontal and corresponding vertical accelerations. The securing arrangement has to be designed to withstand the forces due to acceleration in each horizontal direction (longitudinal and transverse) separately [75,76]. Illustration of applicable acceleration coefficients for road transport is shown on Figure 2.

### 3.3. Evaluation of the Measured Data

It was necessary to evaluate the raw data measured with sensors prior to their assessment and interpretation. According to the standard EN 12195-1:2010 [75], with superposition of the weight of the load, high-frequency stresses and occasional shock loadings of short duration are absorbed by elongation of the lashing devices and the shock absorber systems of lorries and trailers. This occurs without any significant increase in stress; therefore, this can be ignored for the purpose of this European Standard which gives a practical and not a scientific view [75]. This standard is used for road inspections of cargo securing in the European Union according to the Directive 2014/47/EU of the European Parliament and of the Council of 3 April 2014 on the technical roadside inspection of the roadworthiness of commercial vehicles circulating in the Union [77] as well as for the transport of dangerous goods in subsection 7.5.7.1 of the Agreement Concerning the International Carriage of Dangerous Goods by Road (ADR) [78]. Therefore, the aim of the paper is not to study transport shocks, but minimum/maximum average accelerations in periods of 80, 300, and 1000 ms. Maximum/minimum average acceleration in 80 ms is applied in relation to the EN 12642:2016 [79] standard, which is used in strength tests of superstructures or cargo securing by dynamic driving tests.

**Figure 3:**
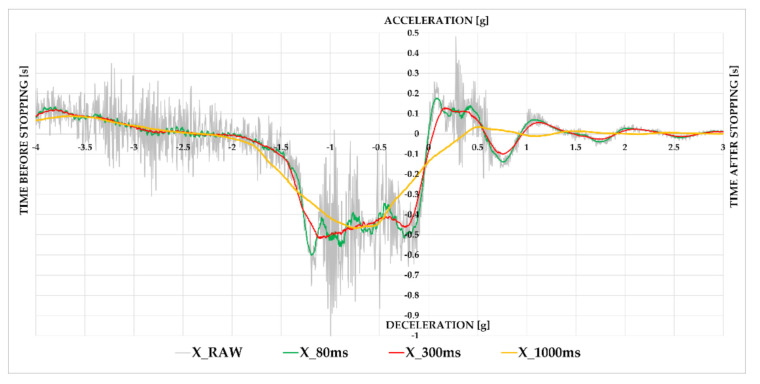
Effect of evaluation time on the course of braking obtained from sensor B during a semi-trailer’s braking.

According to the standard prEN 17321:2020 [80], when dynamic acceleration tests are performed, the minimum deceleration dwell time should be 300 ms. The test value is the minimum acceleration recorded during the dwell time. When dynamic driving tests are performed, the minimum acceleration dwell time should be 1000 ms. With a duration of 80 ms, the arithmetic average should meet the required acceleration value. The arithmetic average of the required acceleration is allowed to fall below the required value by 0.05 g in case this value is applied over a period of one second [75,76]. Following this, the resultant raw values were evaluated using the moving average time periods of 80, 300 and 1000 ms. The visible effect of evaluation time on the course of a semi-trailer’s braking can be seen on Figure 3.

### 3.4. The Methodology of Performed Measurements

#### 3.4.1. A Comparison of Results Measured with Sensors during Braking

In the first phase of the research, it was necessary to verify to what extent the values measured with different sensors correspond to each other. For this reason, we chose to test sensors in one place on one holder, where the sensors were placed close to each other (parallel to the *Y*-axis), and we measured the acceleration in the direction of the *X*-axis, i.e., in the driving direction. Due to practical reasons, we measured the acceleration (or deceleration) in a passenger car of M1 category, where higher measured values of braking deceleration are anticipated compared to trucks. At the same time, in the case of acceleration, lower energy claims are put on a vehicle of M1 category, which results in lower fuel consumption and a smaller volume of emissions produced during the testing.

#### 3.4.2. The Usage of Sensors during Braking with a Loaded Vehicle Combination

After the sensor verification, we took measurements with a loaded semi-trailer combination. The tested vehicles were standard, widely used European semi-trailer vehicle combinations of a 2-axle tractor and a 3-axle curtainsider semi-trailer, 16.5 m long with a 13.6-m-long loading platform.

**Figure 4:**
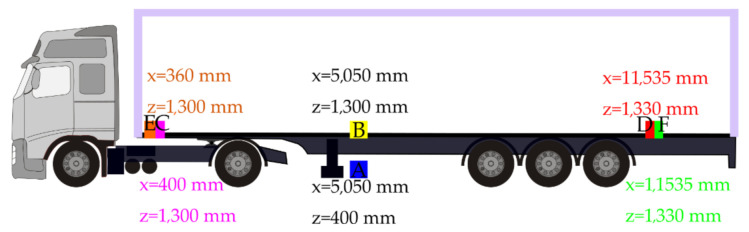
Sensor positions in case of Series 1 braking tests.

The positions of the sensors in relation to the loading platform and road surface, as shown in the example for Series 1 (Figure 4), are indicated in the Supplementary Materials for each test series. Sensors were fixed to the semi-trailer chassis or loading floor by magnets. Straight-line motion in the direction of the *X*-axis was measured as well; however, with such a difference, sensors were placed in different parts of the semi-trailer so that it would be possible to compare acceleration values in different parts of the semi-trailer. Real loadings were chosen in order to simulate various emergency braking of a loaded vehicle combination. More than 120 braking tests were realized (where packaging or cargo was damaged several times) with the following kinds of cargo in the following series:

- Series 1—offset paper, total cargo mass 19,636 kg, (Sensors A–F), vehicle comb. 1;
- Series 2—offset paper, total cargo mass 19,636 kg, (Sensors A–D), vehicle comb. 2;
- Series 3—steel bars, total cargo mass 22,766 kg, (Sensors A–D), vehicle comb. 3;
- Series 4—steel bars, total cargo mass 23,772 kg, (Sensors A–D), vehicle comb. 3;
- Series 5—steel bars, total cargo mass 15,169 kg, (Sensors A–D), vehicle comb. 3;
- Series 6—steel bars, total cargo mass 24,062 kg, (Sensors A–D), vehicle comb. 3;
- Series 7—steel bars, total cargo mass 9841 kg, (Sensors A–D), vehicle comb. 3);
- Series 8—steel bars, total cargo mass 15,142 kg, (Sensors A–D), vehicle comb. 3;

#### 3.4.3. The Usage of Sensors during Monitoring a Semi-trailer Vehicle Combination Transport

In the third phase of the research, we focused on monitoring real transport with semi-trailer combination No. 4. In the transport monitoring, we measured acceleration in all three axes (directions) in a selected transport session. We evaluated maximum values measured with sensors, where we assumed lower acceleration values in comparison to the previous phase with regard to a smooth course of transport (without any emergency braking, avoidance maneuvers, etc.). The measured values were processed into box plot charts and plotted onto a map, which allowed us to identify whether there was an extreme value of acceleration in a section in any directions.

**Figure 5:**
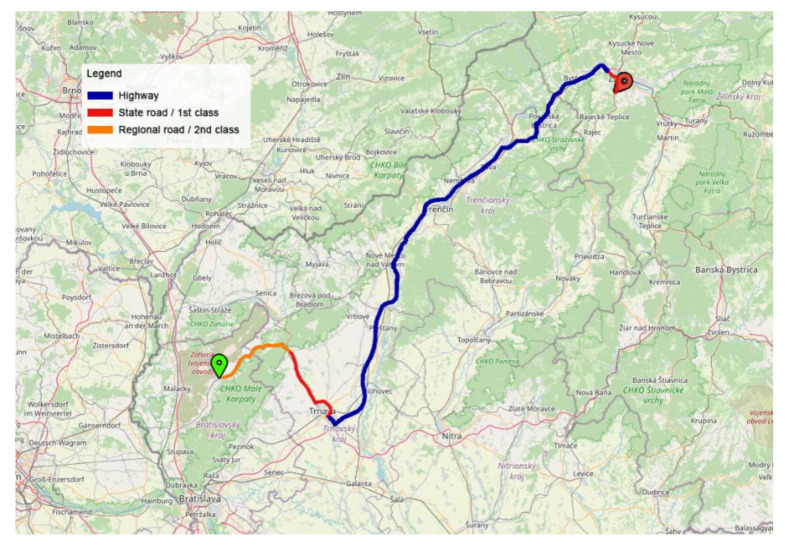
A monitored transport route (map by OpenStreetMap).

We monitored the selected transport of a semi-trailer combination with 17 pieces of pallet load units with bags with a gross cargo weight of 23,800 kg; the transport was monitored using two sensors (sensors C and D). Sensor C was placed in the back part of the semi-trailer (12,160 mm from the vehicle front), and it was positioned on a pallet at a height of 2550 mm above the road. Sensor D was placed in the loading area of the semi-trailer, 7000 mm from the semi-trailer front, and the height was 1300 mm above the road. The transport route in the territory of the Slovak Republic was 211 km long, of which 12% consisted of 2nd class roads (26 km), 17% was 1st class roads (36 km), and the longest transport distance (71%) was realized on a motorway (149 km), as visualized in Figure 5.

## 4. Results

In this section, we will focus on the interpretation of the measured results from the three research phases. Besides the maximum acceleration values in given directions, we also measured other indicators obtained using sensor A, described in 3.1.1.

### 4.1. A Comparison of Results Measured with Sensors during Braking

At first, we compared selected sensors placed close to each other on one holder positioned in the passenger car while focusing on minimum acceleration values in the direction of the *X*-axis during emergency braking. Moreover, we also measured other parameters of braking such as braking distance, braking time, MFDD, and initial braking velocity from all sensors.

Then, when comparing the sensors’ measurements during the passenger car braking, we first concentrated on the comparison of the measured initial braking velocity. We can observe that the values of the vehicle velocity obtained from individual sensors are very similar, except for sensor A. This is because unlike the other MEMS sensors which received velocity data from a GPS chip, sensor A received velocity data via a calculation from the acceleration (deceleration) values.

To evaluate the sensors, we calculated the average velocity for each measurement, based on which we calculated deviations of individual sensors. The deviation from the average velocity is very small; the smallest value was obtained in measurement 4 (sensor D, deviation: −0.73%). Sensors B to E feature almost matching values in the measured velocity since the differences are minimal (approximately tenths to hundredths of km/h).

**Figure 6:**
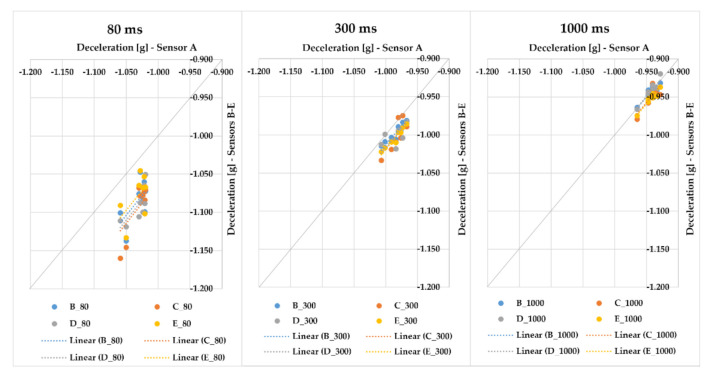
Point dependency of individual microelectromechanical system (MEMS) sensors against sensor A with evaluation times application.

In the next step, we compared the measured maximum values of braking deceleration. The effect of the applied evaluation times on the measured values represented in Figure 3 is confirmed in Figure 6. With the gradual application of evaluation times, the points approached the quadrant axis, which means that the measuring sensors are more alike.

In Table 3, the minimum, average, and maximum deviations and the median are compared between the given pairs of sensors; the 95th percentile of deviations and the dependency between two sensors are expressed with a gradient of a linear trend and a coefficient of determination R^2^. In Table 3, we have highlighted all deviations exceeding the value of 0.03 g. On that basis, we can conclude that differences greater than the set value of 0.03 g mostly occurred only in the case of an 80-ms period and mostly while comparing sensor A to other sensors; this was due to the hardware data filtering performed by sensor A, while the other sensors do not feature such a filter. As for the 80-ms time the greatest deviation occurred between sensors A and C (deviation: 0.101 g; 95th percentile: 0.099 g); if sensor A is excluded from the comparison, the greatest difference can be seen between sensors C and E (deviation: 0.069 g; 95th percentile: 0.057 g). When looking at 300 and 1000 ms, we may say that the deviations among sensors are minimal. In the case of the 300-ms period, the maximum difference, i.e., 0.034 g (95th percentile: 0.033 g) occurred between sensors A and E. If sensor A is excluded from the comparison, the maximum deviation was 0.021 g (95th percentile: 0.018 g) for the pairs B–C and B–D; for the pair C–D, the maximum deviation was 0.029 g (95th percentile: 0.026 g). When 1000 ms was applied, the greatest deviation of 0.027 g (95th percentile: 0.022 g) occurred between sensors C and D. The relation between two sensors expressed with a gradient of a linear trend means that the value measured with one sensor is, for example, 1.0513 times greater than the value measured with another sensor, with the probability of 99.96% (example coming from A–B, 80 ms time).

With respect to the range of the paper, the complete results to compare the sensors, including the braking distance *s_0_*, braking time *T_br_*, MFDD, initial braking velocity *v_0_*, maximum values of braking deceleration, velocity, and average deviations, are processed in Tables S1 and S4, which can be found in the Supplementary Materials.

### 4.2. The Usage of Sensors during Braking with a Loaded Vehicle Combination

**Figure 7:**
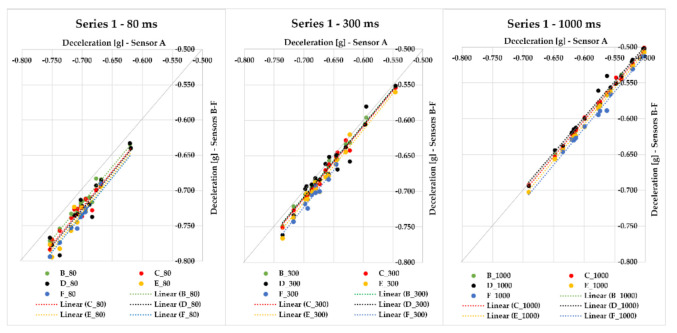
Series 1-Point dependency of individual MEMS sensors against sensor A.

**Figure 8:**
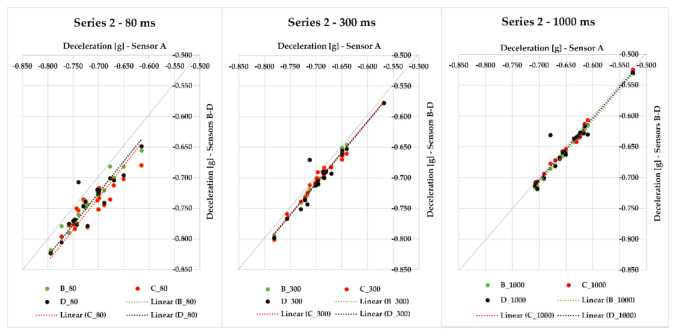
Series 2-Point dependency of individual MEMS sensors against sensor A.

**Figure 9:**
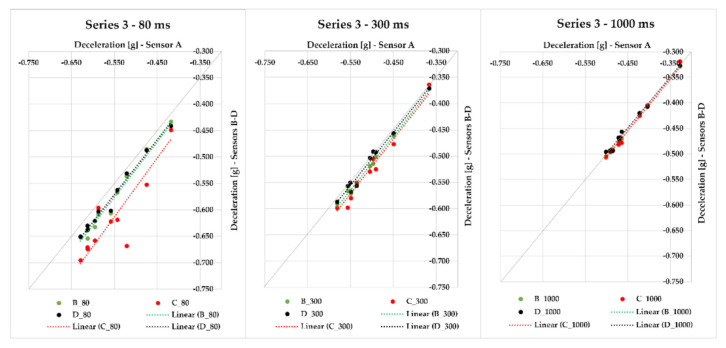
Series 3-A Point Dependency of Individual MEMS Sensors against Sensor A.

**Figure 10:**
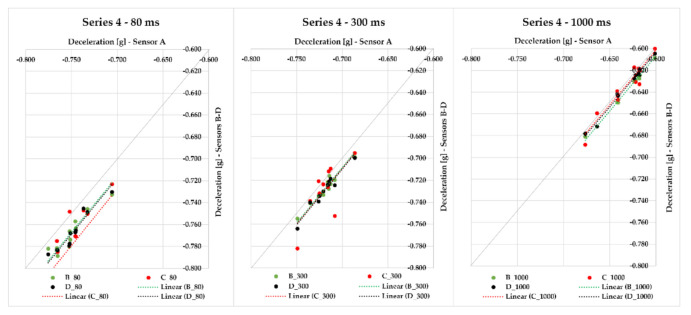
Series 4-Point dependency of individual MEMS sensors against sensor A.

**Figure 11:**
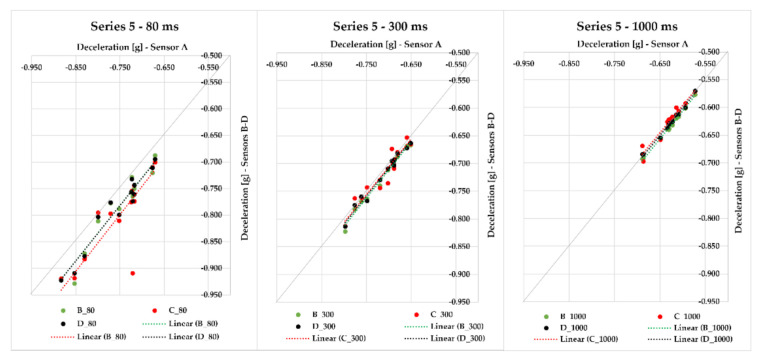
Series 5-Point dependency of individual MEMS sensors against sensor A.

**Figure 12:**
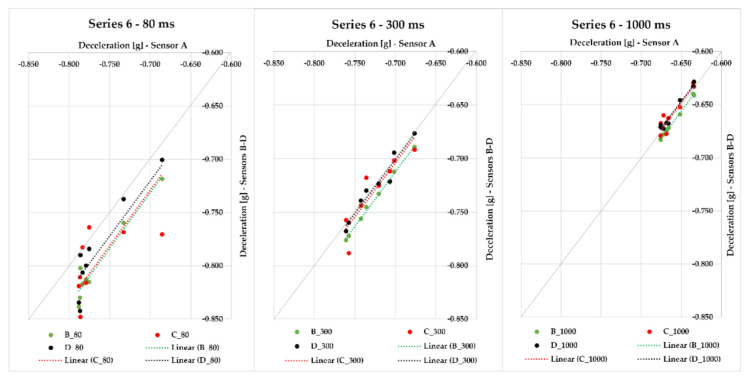
Series 6-Point dependency of individual MEMS sensors against sensor A.

**Figure 13:**
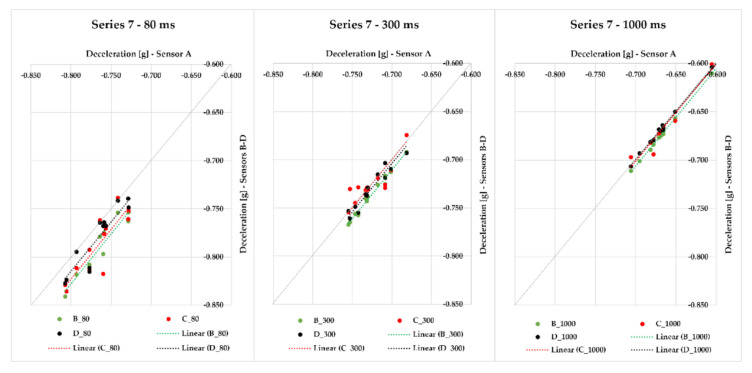
Series 7-Point dependency of individual MEMS sensors against sensor A.

**Figure 14:**
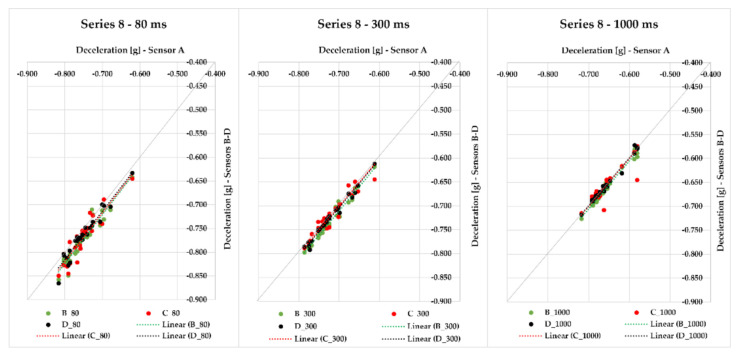
Series 8-Point dependency of individual MEMS sensors against sensor A.

In this part of the research, we focus on braking with a loaded vehicle combination. The results of several test series with different cargo loadings and different positions of sensors placed on a semi-trailer combination are presented in Figure 7, Figure 8, Figure 9, Figure 10, Figure 11, Figure 12, Figure 13 and Figure 14. Figure 7 represents the point dependency of individual MEMS sensors against sensor A in the case of Series 1, where 20 braking events were performed with cargo. This series was realized with sensors A to F, where pallet load units with offset paper characterized by a gross cargo weight of 19,636 kg were loaded on the semi-trailer. It is true that the closer the points are to the quadrant axis (a grey line), the more the sensors are alike. Additionally, in this case of evaluation time application, we can see how the deviation from the average value changes. The differences among the sensors, even though they are very small in principle, are caused by the fact they were placed in different places of the loading area on the vehicle. The greatest deviations against the average value were, again, achieved with sensor A, the cause of which is that sensor A automatically filters the measured raw data using its hardware, whereas other sensors do not feature such a hardware filter. Nevertheless, the differences among sensors are only approximately in hundredths, or tenths of g (the range is from 0.008 to 0.152 g in the case of 80 ms; from 0.006 to 0.068 g in case of 300 ms; and in case of 1000 ms, the range of maximum deviation reached values from 0.001 to 0.025 g). The greatest value of MFDD was obtained during braking ID 4 (ID–identification number), when the mean fully developed deceleration reached the value of 6.39 m/s^2^. 

As part of Series 2, we were braking with a loaded semi-trailer combination (palletized offset paper) with a gross cargo weight of 19,636 kg, with the difference that sensors A–D were used. Altogether, 19 braking events were realized. With the application of each time of 80, 300, and 1000 ms included in Figure 8, we can see how the values approach the quadrant axis, i.e., they are similar. The maximum deviation of sensors against the average value ranges from 0.010 to 0.037 g in the case of 80 ms time; when 300 ms is applied, the maximum deviation ranges from 0.006 to 0.033 g. The time of 1000 ms provides a range of maximum deviation values from 0.003 to 0.037 g. These very small differences among sensors were also caused by the fact that the sensors were placed in different parts of the loading area. In case of braking ID 27, we reached the greatest MFDD value of 6.74 m/s^2^. 

Series 3 differs from the two previous ones in terms of the loaded cargo. The semi-trailer combination was loaded with steel bars with a gross cargo weight of 22,766 kg and was monitored with sensors A–D, which were placed in different places of the loading area. We conducted 10 braking events with the semi-trailer combination in this series. Maximum deviations of the measured deceleration values from the average value ranged from 0.012 to 0.104 g for 80 ms; with gradual application of other times, this difference even decreases—in the case of 300 ms, the maximum deviation ranged from 0.004 to 0.029 g, and for 1000 ms, the maximum deviation differed from the average in the range from 0.002 to 0.007 g, which can also be seen in Figure 9 since the values approach the quadrant axis more closely with an increasing evaluation time. The greatest MFDD value was reached in case of braking ID 5 at 4.92 m/s^2^.

All other series were realized with the same cargo—steel bars. In Series 4, we were braking with the semi-trailer combination 11 times, and the gross cargo weight was 23,772 kg. The data from sensors A–D and their maximum deviations from the average value of braking deceleration were assessed. Since the distances among individual sensors were different, we measured maximum deviations in the range from 0.007 to 0.043 g in the case of the 80-ms evaluation time. The maximum deviation from the average deceleration value with 300 ms evaluation time applied ranged from 0.004 to 0.026 g, and with 1000 ms evaluation time, it ranged from 0.004 to 0.011 g. Figure 10 represents individual values of the braking deceleration and their relation—the closer the values or their linear trends are to the quadrant axis (a grey line), the more they are alike. The measurement ID 11 showed the greatest MFDD value of 6.784 m/s^2^.

In Series 5, we realized other 13 measurements with the application of sensors A–D placed in different positions of the semi-trailer combination’s loading area loaded with cargo (steel bars) of 15,169 kg gross weight. If the moving average 80 ms was applied, the maximum deviation from the average braking deceleration value ranged from 0.009 to 0.117 g; for 300 ms, it was less—the maximum deviation ranged from 0.004 to 0.021 g. The application of 1000 ms resulted in the range of maximum deviations from the average braking deceleration value being from 0.004 to 0.015 g, which are very small differences (Figure 11). It is necessary to emphasize, again, that the sensors measured the braking deceleration in different places of the loading area. The greatest MFDD value was reached in braking ID 27, when the value of 6.838 m/s^2^ was reached.

Series 6 comprises eight braking events with a loaded semi-trailer combination (steel bars) with a gross cargo weight of 24,062 kg. The braking deceleration was measured with sensors A–D, which were placed in the loading area at different distances from the semi-trailer’s front. The maximum deviation from the average braking deceleration value in 80 ms was in the interval from 0.019 to 0.052 g; the interval decreased with an increasing evaluation time—300 ms featured the maximum deviation interval from 0.007 to 0.019 g, and when 1000 ms was applied, the range of maximum deviations from the average braking deceleration value was from 0.005 to 0.011 g; this effect can also be seen in Figure 11 since the points approach the quadrant axis. The greatest MFDD value (6.746 m/s^2^) was reached in braking ID 37.

In Series 7, we realized 12 measurements with loaded steel bars on the semi-trailer combination with a gross cargo weight of 9841 kg. We monitored the maximum braking deceleration values obtained via sensors A–D, which were placed in different places of the semi-trailer’s loading area. We detected the range of deviations from the average braking deceleration value for variants with the application of different evaluation times—in the case of 80 ms, the interval of maximum deviations was from 0.009 to 0.055 g. The application of 300 ms modified the interval of maximum deviations to the range from 0.005 to 0.022 g, and the greatest effect was reached with 1000 ms, which can also be seen in Figure 12. The range of maximum deviations from the average braking deceleration value is from 0.004 to 0.008 g. The greatest MFDD value (7.035 m/s^2^) was reached in braking ID 47. 

Series 8 comprises up to 29 measurements of braking deceleration with loaded steel bars on the semi-trailer combination with a gross weight of 15,142 kg. Additionally, in this case, we applied sensors A–D, which were placed into various positions of the loading area. When an 80-ms time was applied, the interval of maximum deviations from the average value was from 0.010 to 0.037 g; with gradual application of the following evaluation times, this interval decreased (Figure 14). The interval of maximum deviations for the 300-ms time period ranged from 0.003 to 0.023 g, and for 1000-ms time, the interval was from 0.001 to 0.046 g. The greatest mean fully developed deceleration was reached in the measurement ID 56 (MFDD equal to 6.937 m/s^2^).

Regarding the range of the paper, the complete results for all series, including the braking distance *s_0_*, braking time *T_br_*, MFDD, initial braking velocity *v_0_*, maximum values of braking deceleration, gross weight, and the center of gravity of the cargo, the positions of each sensor, and average deviations are processed in Table S2, which can be found in the Supplementary Materials. 

### 4.3. The Usage of Sensors during Monitoring a Semi-Trailer Combination Transport

Since it would be challenging to assess the monitored transport in a session with the length of 211 km as a whole, the route was divided into 18 isochronal sections (Supplementary Materials Figure S3), except for Sections 4 and 10, where the cargo and sensors were controlled, and Section 18, which was shorter due to being the end of the route. The route was monitored with sensors C and D. Each section was evaluated in parts. First of all, individual courses of acceleration and deceleration for each axis as well as the velocity of the vehicle were monitored. Using the courses, we assessed the maximum values of acceleration and deceleration for all axes and for each sensor. In the case when 80 ms time was applied on the *X*-axis, the greatest deceleration (minX80) was measured as −0.389 g in the section C15, and the greatest acceleration (maxX80) had a value of 0.303 g in the section C16. On the *Y*-axis, we reached the greatest deceleration (minY80) in the section C2 (−0.730 g) and the greatest acceleration (maxY80) in the section C14 (0.714 g). The greatest deceleration on the *Z*-axis (minZ80) occurred in the section C9 with a value of −0.549 g, and the greatest acceleration on this axis (maxZ80) was in the section C16 with a value of 0.668 g. Such great values are, however, in the case of a time period of 80 ms, unimportant for the cargo since these are very short-term actions. 

When 300 ms was applied, on the *X*-axis, we reached the greatest deceleration (minX300) in the section D18, when the value −0.178 g was measured. The greatest acceleration in the direction of the *X*-axis (maxX300) in this case was observed in the section D14 (0.230 g). On the *Y*-axis, in a 300-ms period, the greatest deceleration value (minY300) −0.541 was measured in the section C4 and the greatest acceleration value (maxY300) of 0.375 g in the section C2. The maximum deceleration on the *Z*-axis (minZ300) of −0.349 g occurred in the section C15, and the maximum acceleration (maxZ300) with a value of 0.356 g occurred in the same section.

From the point of view of effects acting on the cargo, the most important evaluation time is the moving average of 1000 ms since it provides information on the acceleration or deceleration effect in a longer-lasting moment. On the *X*-axis, it was −0.169 g in the section D1 in the case of deceleration (minX1000) and 0.226 g in the section D14 in the case of acceleration (maxX1000). When this time was applied on the *Y*-axis, the greatest deceleration (minY1000) was measured at −0.292 g in the section C2, and the greatest acceleration (maxY1000) had a value of 0.269 g in the section C1. The maximum deceleration value on the *Z*-axis (minZ1000) was −0124 g in the section C2, and the maximum acceleration (maxZ1000) value of 0.092 g occurred in the same section.

If values for all evaluation times are similar, it means that a long-term action of acceleration or deceleration occurred, acting on the cargo. For example, such an action can be seen in Sections 14 and 7.

All outcomes from each section of the monitored transport route are presented in Table S3, which can be found in the Supplementary Materials.

In the following section, we concentrate on the representation of data from individual sections using box plot charts. A box plot chart is used for graphical visualization of numeric data using their quartiles. Such charts were created for all axes and evaluation times in each section.

Then, values of medians, quartiles, and 5th and 95th percentiles were exported from box plot charts for all sections and both sensors.

Based on the exported data, we figured out that for the *X*-axis, the values of median range from −0.007 to −0.031 g (the average median was equal to −0.018 g), whereas on the *Y*-axis, the values of median belong in the interval from −0.017 to +0.008 g (the average value was equal to −0.003 g). The median for the *Z*-axis belongs in the interval from −0.003 to 0.014 g (the average was equal to 0.004 g). All exported data from the box plot charts are presented in Table S3, which can be found in the Supplementary Materials.

## 5. Discussion

In the first part of the research, in order to verify the sensors, we performed a series of braking tests in a passenger car where all sensors were placed close to each other; we were interested in the values of acceleration (deceleration) on the *X*-axis, i.e., in the driving direction of the vehicle. During the evaluation, we applied three evaluation times as the moving average—80, 300, and 1000 ms. In the analysis of differences among sensors for 80 ms, the maximum difference among sensors was 0.108 g. However, the durations of 300 and 1000 ms are more important since they point out long-term dynamic actions which affect the vehicle and the cargo. With these evaluation times, the maximum deviation was 0.029 g for 300 ms and 0.015 g for 1000 ms, which can be considered minimal differences when speaking about braking with a passenger car, where higher accelerations (decelerations) are anticipated.

In the second part of the research, during braking tests, we also concentrated on the values of acceleration (deceleration) on the *X*-axis, i.e., in the driving direction of the vehicle. The same three evaluation times were applied as in the first part. Following Regulation No. 13 of the Economic Commission for Europe of the United Nations (UN/ECE) [68], the MFDD value must reach at least 5 m/s^2^, which was achieved in all cases when the vehicle was loaded. The exception was Series 3, where the MFDD value ranged from 3.11 to 4.92 m/s^2^. This was caused by inappropriate conditions during braking—the surface of the road on which we conducted these measurements was smooth and dusty at the same time, which impacted the braking effect negatively. 

**Figure 15:**
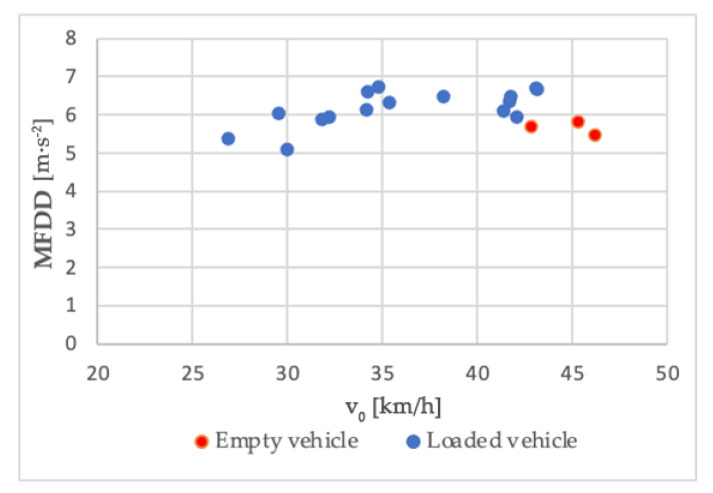
A comparison of mean fully developed deceleration (MFDD) in the case of a loaded and an empty vehicle.

One of the partial results in this part of the research was the finding of the difference in the measured values when we compared a loaded vehicle to an empty vehicle with an unchanged positioning of sensors. This effect can be seen, for example, in Series 8 of the braking tests, where we also conducted three measurements with an empty vehicle at the end. The comparison was performed as follows: we plotted the point dependency of MFDD values (on the *Y*-axis of the chart) on the values of the initial braking velocity (Figure 15). The chart shows that in case of an empty vehicle, the MFDD value is around 5.5 m/s^2^ with the initial velocity from 42 km/h.

Measurements with an empty combination were carried out in a minimum way to determine the braking intensity of the empty vehicle combination. Given that this article deals with the use of MEMS sensors to evaluate accelerations concerning cargo securing, we will not consider the values measured with an empty vehicle combination in the manuscript. However, we can see that the MFDD values achieved with the empty combination are relatively lower than the MFDD values with the loaded combination. Without further testing, it would be incorrect to make conclusions given the low number of measurements with an empty combination. As one possible explanation of why the MFDD value achieved with an empty vehicle combination is lower than that with a loaded combination, we can refer to the design of the function of the brake system of the semi-trailer. 

The pressure distributed to the brake cylinder circuit in pneumatically sprung trailers is controlled based on the pressure in the pneumatics springs by pressure modulators. In the semi-trailer used during the tests, the pressure in the air suspension circuit in the empty state reached 20 kPa, which corresponds to the maximum pressure acting in the brake cylinders *p_zyl_* = 140 kPa at brake control pressure *p_m_* = 650 kPa. At full axle load (9000 kg/axle), the pressure in the pneumatic springs reached 530 kPa, which corresponds to the maximum pressure in the brake cylinders *p_zyl_* = 600 kPa at the control pressure *p_m_* = 650 kPa. The increase in pressure in the brake pipe is constant with empty and loaded semi-trailers in the pressure range *p_m_* ≤ 60 kPa. At *p_m_* = 60 kPa, the pressure in the brake cylinders reached the value *p_zyl_* = 40 kPa. In the range *p_m_* = (60; 650) kPa, we observed a significant difference in the pressure distributed to the brake cylinders.

While with an empty trailer, at a pressure value of *p_m_* = 650 kPa, the pressure in the brake cylinders reaches the value *p_zyl_* = 140 kPa, with a fully loaded semi-trailer, it is *p_zyl_* = 600 kPa. Due to the gradual increase in *p_zyl_* pressure in the range *p_m_* = (60; 650) kPa, it is difficult to correct the distribution of the optimal *p_zyl_* value for achieving the maximum braking deceleration, despite the electronic control. Braking can be, to a greater extent, negatively affected by minimal road unevenness when, at higher pressure *p_zyl_*, there is braking of individual wheels and the subsequent intervention of Antilock Braking System (ABS) or, at lower pressure, the braking force is not sufficient to fully utilize the tire’s ability to transmit longitudinal forces.

To demonstrate the differences in the measured values from different sensors located in different places of the loading area, we interpreted data from all measurements. In the analysis of these data, we expressed the minimum (MIN), average (AVG), and maximum (MAX) deviation values, and the median within the pair was compared; the dependency between two sensors was expressed with a gradient of a linear trend and a coefficient of determination R^2^. The complete results can be found in Supplementary Table S4.

In Table 4, we summarize the maximum and the 95th percentile values of deviation which arose among the values measured with individual sensors for measurements with a passenger vehicle and all eight series of measurements with semi-trailer combinations. The values which exceeded the limit of 0.03 g are marked with a red dot. The greatest deviations emerged when a time of 80 ms was applied; the limit of 0.3 g was exceeded up to 47 times in 54 values. The smallest deviation emerged between sensors B and D during the fourth series of braking, and it reached the value of 0.011 g. On the contrary, the greatest deviation—specifically, the value 0.188 g—was achieved during Series 5 between sensors A and C. In Series 5, we may observe significant deviations mainly in values measured with sensor C when compared to values measured with other sensors, which could have been caused by the deflection of the frame of the semi-trailer. A significant deviation in values measured with sensor C is also observed in Series 3. The values of the 95th percentile range from 0.011 g for sensors B and D during Series 4 up to 0.116 g between sensors A and C during Series 3. When a time of 80 ms was applied, the maximum values of deviations measured with individual sensors ranged from 0.009 g between sensors B and D during Series 4 up to 0.048 g between the same sensors. However, during Series 2, the set limit 0.03 g was exceeded 15 times in 54 values, which represents a reduction in the number of exceeding cases to approximately one-third when compared to the result when a time of 80 ms was applied. While applying the 95th percentile, the value 0.03 g was exceeded six times only, and the values of the 95th percentile ranged from 0.007 g for sensors B and D during Series 4 up to 0.039 g between sensors A and C during Series 4. When a time of 1000 ms time was applied, the limit 0.03 g was exceeded in the case of maximum deviations and the 95th percentile of deviations only for Series 8 between sensors A and C, B and C, and C and D. The limit 0.03 g was exceeded only in the case of maximum deviation values; for the 95th percentile, the deviation was below 0.03 g. Since there is an unusually high value of deviation in these series only in relation to one sensor (sensor C in Series 8 and sensor D in Series 2), it may be anticipated that this deviation is a result of the semi-trailer’s deflection. The maximum deviation values ranged from 0.003 g between sensors A and D during Series 7 up to 0.065 g between sensors C and D during Series 8. The values of the 95th percentile ranged from 0.003 g between sensors A and D during Series 7 up to 0.035 g between sensors A and C, B and C, and C and D during Series 8.

In the research, we also focused on the identification and analysis of “exceptional event” occurrences during transport. The first step was to define the level of acceleration on individual axes; in case it was exceeded, the event would be classified as an “exceptional event”. With respect to the analysis of accelerations given above, we worked out a multi-level division of “exceptional events”, and the following values were chosen for the limits: 0.3, 0.5, 0.6, and 0.7 g, provided that among individual events, there must be a time span of at least 2 s to eliminate cases where one action would be evaluated as multiple “exceptional events”.

**Figure 16:**
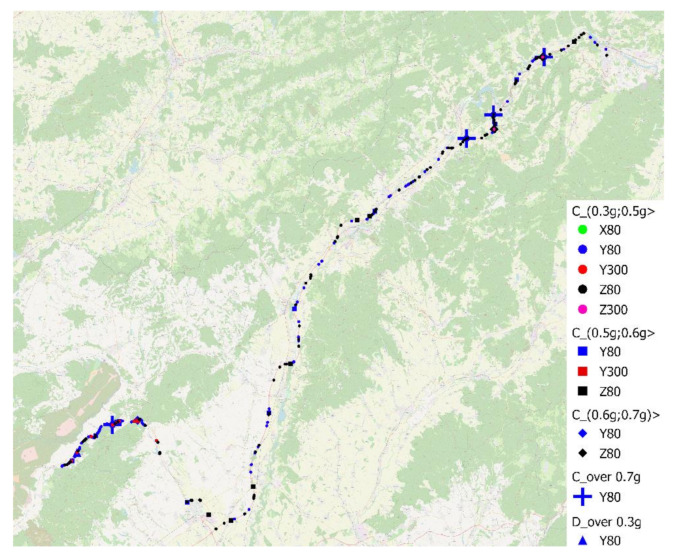
Exceptional events during transport (map by OpenStreetMap).

Then, the corresponding coordinates were marked into a map which resulted in a graphical representation. The illustration map can be seen in Figure 16; a more detailed version is provided as part of Supplementary Figure S1, and the details of Sections 1–3 can be found in Figure S2.

The graphical representation, however, does not offer the option to explore the occurrence of “exceptional events” more closely; therefore we also analyzed the obtained data in a table format, presented in Supplementary Table S5.

The table shows that in the case of sensor D, only one “exceptional event” was recorded; namely, it was the exceeding of the acceleration limit at the level of 0.3 g in the direction of the *Y*-axis while a time of 80 ms was applied. This event happened in the first section out of the 18 monitored ones. In the case of sensor C, there were 352 events recorded altogether (all axes). Of these, 320 events reached the acceleration value in the interval (0.3; 0.5 > g), 25 events reached an acceleration value between 0.5 and 0.6 g, 3 events had an acceleration value in the interval (0.6; 0.7 > g), and 4 events exceeded the limit of 0.7 g.

The majority of events, when the acceleration value was in the interval (0.3; 0.5 > g, were observed in the direction of the *Y*-axis with data evaluated in a time of 80 ms; specifically, 186 events which reached this value were noted, which represents 53% of the 352 events in total. Following these are the events observed in the direction of the *Z*-axis, again with data evaluated in a time of 80 ms; the number of recorded events was equal to 112, i.e., 32%. Furthermore, 5% of events, i.e., 16 events, were detected in the direction of the *Y*-axis from data evaluated in a time of 300 ms; four events were recorded in the direction of the *X*-axis while applying a time of 80 ms, and two events in the direction of the *Z*-axis while applying a time of 300 ms. In the case of data in the direction of the *X*-axis evaluated in 300 and 1000 ms, and in the direction of the *Y*-axis and the *Z*-axis evaluated in a time of 1000 ms, there was no “exceptional event” recorded. Moreover, we analyzed the number of “exceptional events’’ that occurred in individual sections; however, with regard to the different lengths of the sections, it was necessary to relativize the data. In our case, we chose the “number of events per 1 km” as the evaluative indicator. We evaluated the occurrence of events in the direction of the *Y*-axis with 80- and 300-ms evaluation times, and in the direction of the *Z*-axis, with 80 ms time. From the point of view of the “exceptional event” occurrence frequency, the following Sections 1–3 were evaluated as critical sections for accelerations in the direction of the Y-axis; however, for accelerations in the direction of the Z-axis, the critical sections were Sections 1, 10, 14–16. Sections 1–3 comprised the driving mostly on 2nd class roads, whereas Sections 7–17 mostly comprised driving on a motorway. 

While analyzing the number of events in the interval (0.5; 0.6 > g out of the total number of 25, up to 16 events were recorded in the direction of the *Y*-axis with values evaluated to 80 ms time, which creates a 64% share. In this case the critical section is the Section 2, with 0.75 event/km of driving on average. Eight events, which is a 32% share of events in this interval, were recorded in the direction of the Z-axis when evaluated to 80 ms, and these events mostly happened during the driving on a motorway. One event was recorded in the direction of the Y-axis when evaluated to 300 ms, specifically, it was in Section 3.

Out of three events in total when the acceleration in the interval (0.6 g; 0.7 > g was measured, one event was recorded in the direction of the *Y*-axis and two events in the direction of the *Z*-axis, with the values evaluated in a time of 80 ms in both cases.

**Figure 17:**
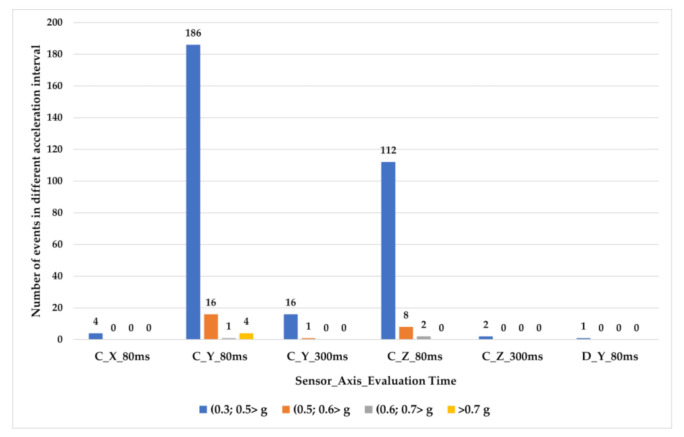
Numbers of events per particular acceleration intervals.

The acceleration limit of 0.7 g was exceeded four times—in each case, in the direction of the *Y*-axis in a time of 80 ms—specifically, in Sections 2, 14–16. During the transport monitoring, there was no intensive deceleration as arises in braking tests, since in this type of transport, it was not necessary to perform emergency sharp braking, e.g., due to an exceptional situation in the road traffic. Number of events per particular acceleration intervals are shown on Figure 17.

Since the individual events shown on the map and evaluated in the Supplementary Materials do not describe the actual events, we decided to analyze these events in a more detailed way and identify the most probable causes of their occurrence.

We primarily focused on the analysis of events exceeding an acceleration value of 0.7 g. This value was exceeded in four cases, specifically within Sections 2, 14– 16. An event in Section 2 occurred on a 2nd class road at a velocity of approximately 40 km/h, with an acceleration value of 0.73 g recorded by sensor C in the direction of the *Y*-axis in 80 ms. In the analysis of a specific event, we found that the acceleration achieved in 300 ms was 0.25 g, so when applying a 300-ms evaluation time, we obtain approximately one-third of the acceleration value compared to 80 ms, which indicates that the sensor is affected by short-term events. Given that no other axis shows an acceleration great than 0.1 g during this event, and, at the same time, the highest acceleration value was also measured in the direction of the *Y*-axis by sensor D (only 0.116 g in 80 ms), we can assume that in this case, the event occurred due to the running of one axle of the semi-trailer on uneven road.

Another three events exceeding the acceleration value of 0.7 g occurred when driving on the highway at a speed of 85–87 km/h. Again, the limit value was exceeded in sensor C along the *Y*-axis using an 80-ms evaluation time. In all cases, events occurred at the beginning of bridges, and it is possible to surely assume that their cause was passage through the dilatation on the road. At the same time, a more significant change in acceleration along the *Z*-axis was observed during these events, the values of which ranged from 0.15 to 0.21 g. We also analyzed the immediate location of these events and found that in these areas of the route, extreme values of acceleration were achieved for a given route section, especially in the direction of the X- and Y-axes when applying 80- and 300-ms evaluation times, but none of the values exceeded 0.3 g. 

A similar situation occurred in the analysis of events reaching the value of acceleration in the interval (0.6; 0.7 > g, of which there were three on the whole route. In one case, acceleration greater than 0.6 g was achieved in the direction of the Y-axis, reaching a value of 0.611 g in Section 3; in two cases, the value of 0.6 g was exceeded in the vertical direction (Z-axis)—0.601 g in Section 15 and 0.606 g in Section 16 in an 80-ms evaluation time. 

Acceleration values in the interval (0.5; 0.6> g) were reached in 25 cases, while in 16 cases, the limit value was exceeded in the Y-axis; in eight cases, in the Z-axis during 80 ms, and in one case, the limit was exceeded in the Y-axis during 300 ms (0.51 g) in Section 3, and this point also corresponds to the point at which the limit of 0.6 g was exceeded during a period of 80 ms—namely, a value of 0.611 g was reached. We can consider this event as the heaviest dynamics influencing the cargo in the rear part of the semi-trailer on the whole transport route. Since there was only a single exceedance during such a long time, we analyzed the place of occurrence of this event and found out that the road in this part of the leg was significantly damaged, which caused a rapid movement of the semi-trailer in the side direction (tilt), which manifested especially in its rear part where sensor C was located. At this point, in the *Y*-axis, the acceleration value measured by sensor D was 0.184 g for an 80-ms period and 0.159 g for 300 ms. The relatively significant difference in the measured values clearly indicates the different effect of acceleration in different parts of the trailer. No significant acceleration values were observed in this location for other axes.

The results indicate that even though in several cases, the limit of 0.5 was exceeded—which, in relation to EN 12195-1 [74], is considered the minimum value necessary to secure cargo against lateral movement—acceleration was exceeded for a time longer than 300 ms in only one case, and in this case, the value of lateral acceleration was 0.51 g. However, the effect of acceleration on a load for less than 300 ms does not affect securing against the movement. Although the standard considers the need to secure the cargo against forward movement with a force equal to 80% of the cargo (which corresponds to 0.8 g acceleration), during transport, acceleration in the *X*-axis above 0.5 g was not exceeded even once, while in four cases, the value was greater than 0.3 g. In neither case was there a change in velocity, except when crossing over the dilatations of bridges and short-term events, since in no case during the time of 300 ms was the achieved value of acceleration greater than 0.14 g, or, during a period of 1000 ms, greater than 0.03 g. In the analysis of the location of these events, we also found increased values of acceleration in the *Z*-axis. There were also values of acceleration in the *X*-axis up to 0.08 g in a period of 1000 ms. 

Inertial forces for cargo securing are considered as static; therefore, different standards use different evaluation times for these accelerations as maximum/minimum average acceleration of 80 and 1000 ms for dynamic driving tests and 300 ms for dynamic acceleration tests. The direct raw data of sensors were not used for this purpose. 

A period of 1000 ms is too long to capture short events and it is more suitable for long events such as heavy braking from a higher velocity, long cornering, or change of lane maneuver. For short events, periods of 80 and 300 ms are important. The highest acceleration in 1000 ms is the heaviest dynamic event that affects the cargo. For example, if the pallet units were tested by dynamic acceleration tests with a minimum time of 300 ms, we would know what the maximum/minimum acceleration in 300 ms was during the transport. According to our observations and results analysis, all three evaluation times could be used for evaluation of acceleration to see the effects of long and short dynamic events, which is not the case for the current monitoring equipment applied for transport monitoring. Therefore, the results between different monitoring equipment are not comparable.

The most appropriate positioning of sensors depends on the testing needs. If the acceleration of different parts of a vehicle body or cargo is being studied, sensors should be placed accordingly. The position of the sensors should be always indicated. The cargo in the rear part of a semi-trailer is exposed to the highest acceleration. Here, greater damage to the cargo usually occurs. Therefore, when this cargo needs to be analyzed for damage, sensors should be placed accordingly. When considering transport monitoring, usually, only one sensor will be used and placed on a semi-trailer below the cargo floor, e.g., in the middle of the cargo floor. If abnormal transport is monitored, the use of two sensors is recommended: one on the cargo at the height of the center of gravity of the cargo, and one on the trailer floor, aligned in vertical axis to compare the results from the cargo and the trailer, especially for accelerations in the *Y*-axis.

In Table 5, we present the maximum acceleration and deceleration values measured during transport by sensor C on all three axes (X, Y, and Z). The measured RAW acceleration and deceleration extremes far exceed the values of 1 or 2 g; in the case of the *Y*-axis, the maximum value reached 8 g. However, examining raw data will not give us a realistic view of how accelerations affect the cargo in certain time periods; therefore, we evaluated them according to time periods of 80, 300, and 1000 ms. Extreme raw values above 1 g for the X- and Y-axes or 4 g for the *Z*-axis were also measured by the authors in [81,82,83], who monitored the transport carried out on a highway or unpaved roads. However, the authors used different methods for measuring the acceleration (deceleration) as well as a different approach to process and evaluate the data, as they did not consider the effect of acceleration and forces on the cargo in the specified evaluation times of 80, 300, and 1000 ms.

## 6. Conclusions

In this paper, we aimed to identify dynamic events affecting cargo during road transport in order to increase the safety of road traffic. It is necessary to realize that the dynamics acting on a vehicle and its cargo differ in various parts of the vehicle during transport, and thus, it is required to know these forces and to measure and evaluate them in order to avoid damaging the cargo, vehicle, and infrastructure and/or to prevent health and life risks for the driver and other participants in the road traffic. To identify acceleration or deceleration forces acting on the vehicle, we chose six different MEMS sensors for the measurements; accelerometers and GPS chips were measuring the acceleration (deceleration) and the GPS chips were measuring position and velocity. These MEMS sensors were selected due to their appropriateness for our purpose, i.e., the acceleration values were recorded with a frequency of 200 Hz with the range of ±8 g (±2 g in case of sensor A which was used only for braking tests). The placement of sensors in the loading area plays an important role; thus, the positions of individual sensors were chosen so that they could measure the acceleration in various places of the semi-trailer’s loading area, since the acceleration is not equal in all places of the vehicle.

Initially, we had to verify whether the selected sensors were suitable for measuring acceleration in road traffic and whether the resulting values corresponded with each other in the case of braking tests. From the realized measurements, we concluded that the sensors provided almost identical results, and therefore, they were used for other measurements.

In the second part of the measurement, eight series of braking tests were performed in vehicles with a different cargo and a different number of applied sensors, which were placed at multiple locations in the loading area of a semi-trailer combination. For the evaluation of the measured data, we concentrated on the minimum acceleration in 80, 300, and 1000 ms in the *X*-axis; in addition, to describe the test conditions, we indicated the braking distance, braking time, mean fully developed deceleration, and initial braking velocity. The obtained maximum deceleration values were compared to each other from the perspective of the sensors’ placement in the semi-trailer’s loading area. 

In the case of a different positioning of sensors on the semi-trailer, the maximum deviation among sensors B, C, and D was 0.145 g (95th percentile 0.052 g) in a time of 80 ms. When the sensors were placed in the front and back parts of the semi-trailer, it was 0.079 g (95th percentile 0.076 g) in 80 ms, since the sensor placed in the middle part of the semi-trailer features greater deviations with regard to the semi-trailer’s frame deflection during braking tests. 

In the third part, during the transport monitoring, some significant differences in accelerations measured with sensor C and sensor D were found. These differences were caused by the different positions of sensors in the semi-trailer. Sensor D was placed on the loading platform 7000 mm from the semi-trailer’s front wall at a height of 1300 mm above the road, meaning that it was placed between the kingpin and the triple axle of the semi-trailer. The results make it clear that in this part of the semi-trailer, significantly lower accelerations emerge than in the back part of the semi-trailer where sensor C was also placed, 12,160 mm from the semi-trailer’s front wall and at a height of 2550 mm above the road, i.e., behind the triple axle of the semi-trailer. The heaviest dynamics influencing the cargo were measured in the rear of the semi-trailer in Section 3. The critical value was measured in the *Y*-axis in 300 ms and reached 0.51 g. 

If the triple axle drives on a rough road, a lower deceleration acts on the cargo in the front part of the semi-trailer than on the cargo in its back part. The difference in the sensors’ placements, however, was not in only the longitudinal axis but also in the vertical axis, since sensor D was placed on the floor of the semi-trailer and sensor C was placed on pallet load units 1250 mm above the loading platform. 

Based on the results of our measurements, MEMS sensors can be used for several applications to increase transport safety. From the results, we found that it is necessary to evaluate the data within the time of 80 ms, 300 ms, and 1000 ms to have comparable results with tests according to EN 12642:2016 and prEN 17321:2020 and therefore do not use RAW data that distort the results. MEMS sensors can also be used to measure MFDD for comparison with a reference value defined by Regulation No. 13 of the Economic Commission for Europe of the United Nations (UN/ECE). Using MEMS, we found the difference between the measured values with an empty vehicle and a loaded vehicle without changing the sensors‘ position. During several measurements where we changed the sensor position, we found differences in accelerations in different parts of the loading area and identified the riskiest place in the loading area. We know from the experience that the rear part of the semi-trailer is the place where most cases of damage occur. With MEMS, we have identified exceptional events that occur during the regular transport of cargo, using specified threshold accelerations. If we analyzed these events, we determined the probable cause of their occurrence. We observed events in which the limit of acceleration considered as the minimum value for securing the cargo against lateral movement by the standard EN 12195-1:2010 was exceeded. We examined the duration of the acceleration and the effect on the cargo and the cargo securing.

Although, we assumed a high incidence of events exceeding the defined acceleration values, especially in the initial and final sections of the route due to lower road categories, several exceedances were identified during highway transport. These events mainly occurred at bridge edges (crossing through the dilatations). Based on the presented findings, we can state that it is possible to use MEMS devices not only to monitor cargo for its proper securing, but also to support the process of analyzing the condition of the road network based on accelerations caused by crossing selected sections. For these purposes, in future research, it will be possible to carry out further research with different types of vehicles, comparing the results based on the type of vehicle, the type of suspension, and also the velocity of crossing through selected sections of the road network.

For the sake of further research, it would be appropriate to compare the measured accelerations with different positions of sensors in the semi-trailer. We would like to compare sensors placed in the same position in the direction of the *Z*-axis (placed at the same height) and in a different position on the *X*-axis, or in the same position on the *X*-axis (placed at the same distance from the semi-trailer’s front wall) and in a different position in the direction of the *Z*-axis, i.e., at a different height; furthermore, individual distances among sensors would be graded appropriately.

## Figures and Tables

**Table 1:** The range and sensitivity of the used sensors [67,68,69,70,71,72,73].

	Accelerometer		Gyroscope	
	Range	Sensitivity	Range	Sensitivity
Sensor A	±2 g	±1%	-	-
Sensor B	±8 g	4096 LSB/g	±500 dps	±65.6 LSB/dps
Sensors C and D	±8 g	1024 LSB/g	±500 dps	±65.6 LSB/dps
Sensors E and F	±8 g	4096 LSB/g	±500 dps	±65.6 LSB/dps

Note: LSB-Least Significant Bit/Byte, dps-degrees per second.

**Table 2:** Applicable acceleration coefficients for road transport [75,76].

	Acceleration Coefficients			
Securing in	Longitudinally (*c_x_*)		Transversely (*c_y_*)	Minimum Vertically Down (*c_z_*)
	Forward	Rearward		
Longitudinal direction	0.8	0.5	-	1.0
Transverse direction	-	-	0.5	1.0

**Table 3:** Comparison of differences between sensors during passenger car braking.

	**Deviation of Maximum Average Acceleration in 80 ms [g]**									
	**A–B**	**A–C**	**A–D**	**A–E**	**B–C**	**B–D**	**B–E**	**C–D**	**C–E**	**D–E**
MIN	0.020	0.038•	0.031•	0.018	0.002	0.005	0.002	0.004	0.003	0.012
AVG	0.053•	0.063•	0.059•	0.046•	0.018	0.019	0.007	0.022	0.021	0.024
MED	0.049•	0.052•	0.064•	0.039•	0.011	0.017	0.006	0.021	0.015	0.018
MAX	0.087•	0.101•	0.076•	0.083•	0.060•	0.040•	0.016	0.049•	0.069•	0.042•
y	1.0513x	1.0614x	1.0574x	1.0449x	1.0094x	1.0055x	0.994x	0.9959x	0.9843x	0.9881x
R^2^	0.9996	0.9996	0.9998	0.9996	0.9996	0.9996	1.0000	0.9994	0.9995	0.9995
95th PERC	0.084•	0.099•	0.076•	0.082•	0.050•	0.037•	0.014	0.045•	0.057•	0.042•
	**Deviation of maximum average acceleration in 300 ms [g]**									
	**A–B**	**A–C**	**A–D**	**A–E**	**B–C**	**B–D**	**B–E**	**C–D**	**C–E**	**D–E**
MIN	0.008	0.002	0.002	0.002	0.001	0.001	0.003	0.007	0.000	0..002
AVG	0.013	0.019	0.019	0.017	0.007	0.007	0.006	0.016	0.008	0.008
MED	0.012	0.024	0.024	0.016	0.005	0.005	0.006	0.015	0.008	0.007
MAX	0.022	0.028	0.028	0.034•	0.021	0.021	0.008	0.029	0.019	0.018
y	1.0129x	1.0185x	1.0167x	1.0189x	1.0055x	1.0038x	1.0059x	0.9981x	1.0003x	1.002x
R^2^	1.0000	0.9999	0.9999	1.0000	0.9999	0.9999	1.0000	0.9997	0.9999	0.9999
95th PERC	0.020	0.028	0.028	0.033•	0.018	0.018	0.008	0.026	0.018	0.016
	**Deviation of maximum average acceleration in 1000 ms [g]**									
	**A–B**	**A–C**	**A–D**	**A–E**	**B–C**	**B–D**	**B–E**	**C–D**	**C–E**	**D–E**
MIN	0.000	0.000	0.001	0.004	0.000	0.003	0.005	0.002	0.001	0.002
AVG	0.003	0.009	0.004	0.009	0.009	0.005	0.009	0.011	0.007	0..009
MED	0.003	0.010	0.004	0.009	0.007	0.004	0.009	0.011	0.006	0.010
MAX	0.008	0.019	0.009	0.015	0.017	0.012	0.011	0.027	0.013	0.017
y	0.9997x	1.0077x	0.9991x	1.0092x	1.008x	0.9994x	1.0095x	0.9914x	1.0013x	1.01x
R^2^	1.0000	0.9999	1.0000	1.0000	0.9999	1.0000	1.0000	0.9999	0.9999	1.0000
95th PERC	0.007	0.017	0.009	0.014	0.017	0.011	0.011	0.022	0.012	0.016

The values which exceeded the limit of 0.03 g are marked with a red dot.

**Table 4:** A comparison of maximum deviations and 95th percentiles for sensors A–D and individual series of measurements.

	**Deviation of Maximum Average in 80 ms [g]**											
	**A–B**		**A–C**		**A–D**		**B–C**		**B–D**		**C–D**	
	**MAX**	**95th**	**MAX**	**95th**	**MAX**	**95th**	**MAX**	**95th**	**MAX**	**95th**	**MAX**	**95th**
Car	0.087•	0.084•	0.101•	0.099•	0.076•	0.076•	0.060•	0.050•	0.040•	0.037•	0.049•	0.045•
Series 1	0.033•	0.026	0.044•	0.039•	0.054•	0.054•	0.021	0.018	0.038•	0.025	0.035•	0.023
Series 2	0.053•	0.043•	0.064•	0.060•	0.057•	0.053•	0.054•	0.041•	0.053•	0.040•	0.046•	0.038•
Series 3	0.048•	0.045•	0.147•	0.116•	0.043•	0.035•	0.132•	0.103•	0.017	0.014	0.137•	0.105•
Series 4	0.028	0.027	0.067•	0.064•	0.027	0.026	0.055•	0.048•	0.011	0.011	0.050•	0.045•
Series 5	0.075•	0.056•	0.188•	0.114•	0.056•	0.054•	0.145•	0.076•	0.019	0.014	0.136•	0.073•
Series 6	0.050•	0.048•	0.085•	0.077•	0.056•	0.052•	0.052•	0.052•	0.031•	0.028	0.070•	0.066•
Series 7	0.047•	0.041•	0.095•	0.075•	0.038•	0.036•	0.049•	0.033•	0.029	0.029	0.077•	0.062•
Series 8	0.058•	0.040•	0.054•	0.049•	0.048•	0.036•	0.046•	0.044•	0.035•	0.029	0.053•	0.037•
ALL	0.087•	0.052•	0.188•	0.079•	0.076•	0.056•	0.145•	0.052•	0.053•	0.030•	0.137•	0.057•
	**Deviation of maximum average in 300 ms [g]**											
	**A–B**		**A–C**		**A–D**		**B–C**		**B–D**		**C–D**	
	**MAX**	**95th**	**MAX**	**95th**	**MAX**	**95th**	**MAX**	**95th**	**MAX**	**95th**	**MAX**	**95th**
Car	0.022	0.020	0.028	0.028	0.028	0.028	0.021	0.018	0.021	0.018	0.029	0.026
Series 1	0.013	0.012	0.016	0.015	0.026	0.025	0.010	0.008	0.015	0.015	0.024	0.017
Series 2	0.016	0.014	0.021	0.020	0.042•	0.030•	0.019	0.016	0.048•	0.024	0.041•	0.025
Series 3	0.022	0.020	0.041•	0.038•	0.021	0.020	0.032•	0.028	0.023	0.019	0.041•	0.037•
Series 4	0.013	0.013	0.044•	0.039•	0.016	0.016	0.033•	0.030•	0.009	0.007	0.028	0.024
Series 5	0.025	0.022	0.033•	0.028	0.018	0.017	0.023	0.023	0.010	0.009	0.026	0.025
Series 6	0.015	0.015	0.031•	0.026	0.014	0.012	0.027	0.024	0.018	0.018	0.028	0.024
Series 7	0.017	0.016	0.023	0.022	0.012	0.012	0.034•	0.025	0.014	0.014	0.031•	0.028
Series 8	0.024	0.019	0.033•	0.029	0.019	0.015	0.035•	0.031•	0.018	0.017	0.033•	0.026
ALL	0.025	0.019	0.044•	0.031•	0.042•	0.023	0.035•	0.028	0.048•	0.017	0.041•	0.028
	**Deviation of maximum average in 1000 ms [g]**											
	**A–B**		**A–C**		**A–D**		**B–C**		**B–D**		**C–D**	
	**MAX**	**95th**	**MAX**	**95th**	**MAX**	**95th**	**MAX**	**95th**	**MAX**	**95th**	**MAX**	**95th**
Car	0.008	0.007	0.019	0.017	0.009	0.009	0.017	0.017	0.012	0.011	0.027	0.022
Series 1	0.006	0.005	0.007	0.007	0.006	0.005	0.008	0.006	0.006	0.006	0.009	0.009
Series 2	0.012	0.012	0.011	0.009	0.048•	0.027	0.011	0.007	0.055•	0.025	0.046•	0.029
Series 3	0.007	0.007	0.014	0.012	0.008	0.008	0.010	0.009	0.014	0.013	0.022	0.018
Series 4	0.010	0.009	0.015	0.014	0.006	0.006	0.017	0.015	0.007	0.007	0.017	0.014
Series 5	0.010	0.010	0.020	0.016	0.006	0.006	0.024	0.021	0.009	0.009	0.015	0.014
Series 6	0..007	0.007	0.012	0.011	0.007	0.006	0.018	0.017	0.014	0.014	0.012	0.012
Series 7	0.008	0.007	0.009	0.009	0.003	0.003	0.014	0.012	0.009	0.008	0.010	0.009
Series 8	0.016	0.013	0.064•	0.035•	0.015	0.010	0.056•	0.035•	0.020	0.014	0.065•	0.035•
ALL	0.016	0.010	0.064•	0.014	0.048•	0.011	0.056•	0.019	0.055	0.013	0.065•	0.018

The values which exceeded the limit of 0.03 g are marked with a red dot.

**Table 5:** Maximum accelerations and decelerations in RAW, 80-, 300-, and 1000-ms evaluation times (Authors).

	Min RAW	Max RAW	Min 80	Max 80	Min 300	Max 300	Min 1000	Max 1000
X [g]	−3.632	3.613	−0.389	0.304	−0.178	0.230	−0.169	0.226
Y [g]	−7.969	7.980	−0.730	0.714	−0.541	0.375	−0.292	0.269
Z [g]	−4.082	3.530	−0.549	0.668	−0.349	0.356	−0.125	0.092

## Data Availability

Not applicable.

## Supplementary Materials

The following are available online at: https://www.mdpi.com/article/10.3390/s21082881/s1, Table S1: Worksheets for comparison of sensors on passenger car; Table S2: Worksheets for evaluation of individual braking series with semi-trailer vehicle combinations; Table S3: Worksheets for evaluation of monitored transport with semi-trailer combination; Table S4: Worksheets for comparison of the influence of different position of sensors on measured values; Table S5: Worksheets for evaluation of events on a monitored route with semi-trailer vehicle combination; Figure S1: Events on a monitored route with semi-trailer vehicle combination; Figure S2: Events on a monitored route with semi-trailer vehicle combination-detail (leg 1–3); Figure S3: Division of the monitored route into sections.
