# Supplementary figures and images for: Application of MEMS Sensors for Evaluation of the Dynamics for Cargo Securing on Road Vehicles
Source: Sensors (Basel). 2021 Apr 20;21(8):2881. doi: 10.3390/s21082881 (PMC8074253; doi:10.3390/s21082881)

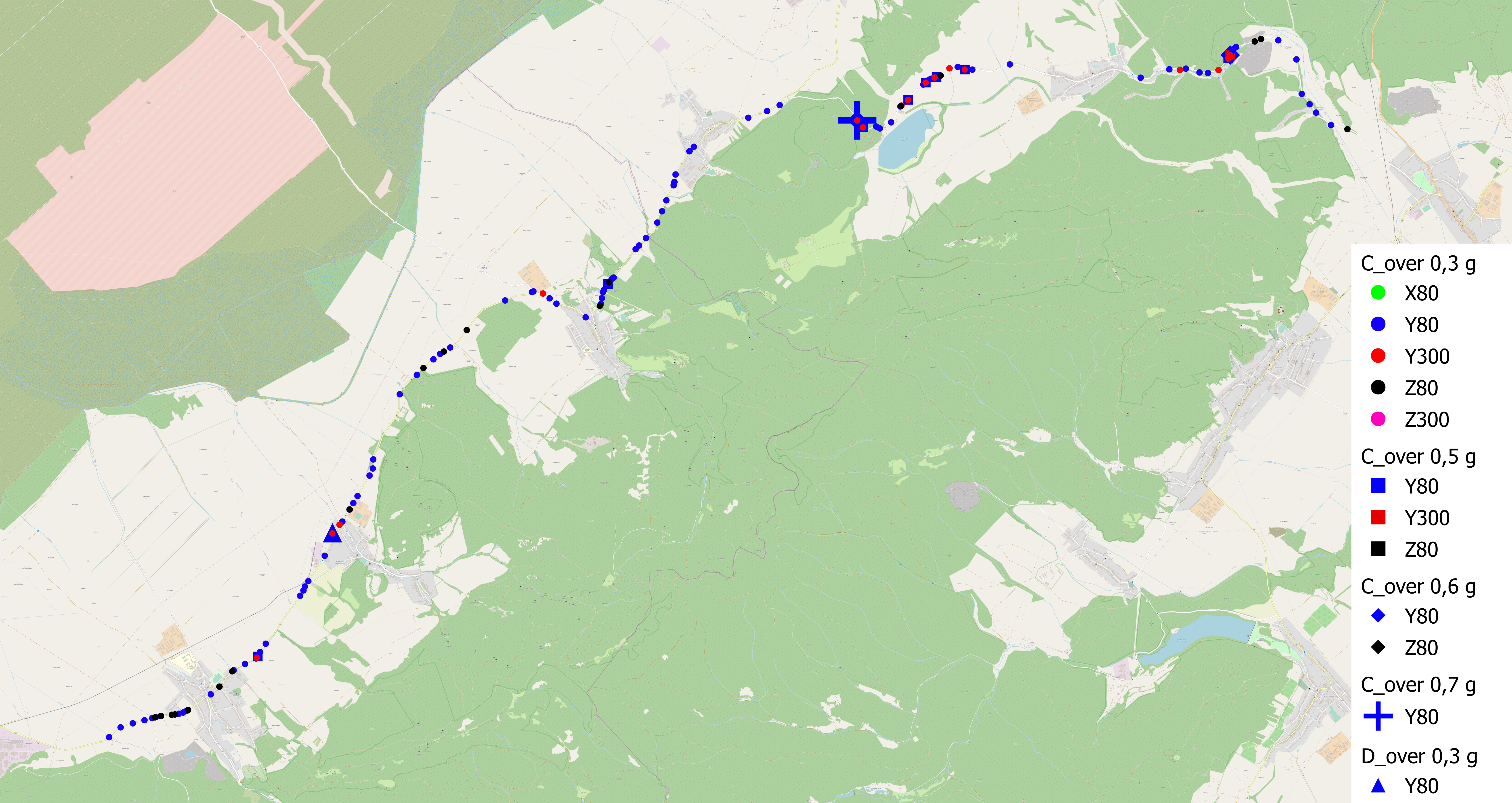

Supplement: Supplementary file 1 [file sensors-21-02881-s001.zip › Figure S2 - Events on a monitored route with semi-trailer vehicle combination - detail (leg 1-3).png]

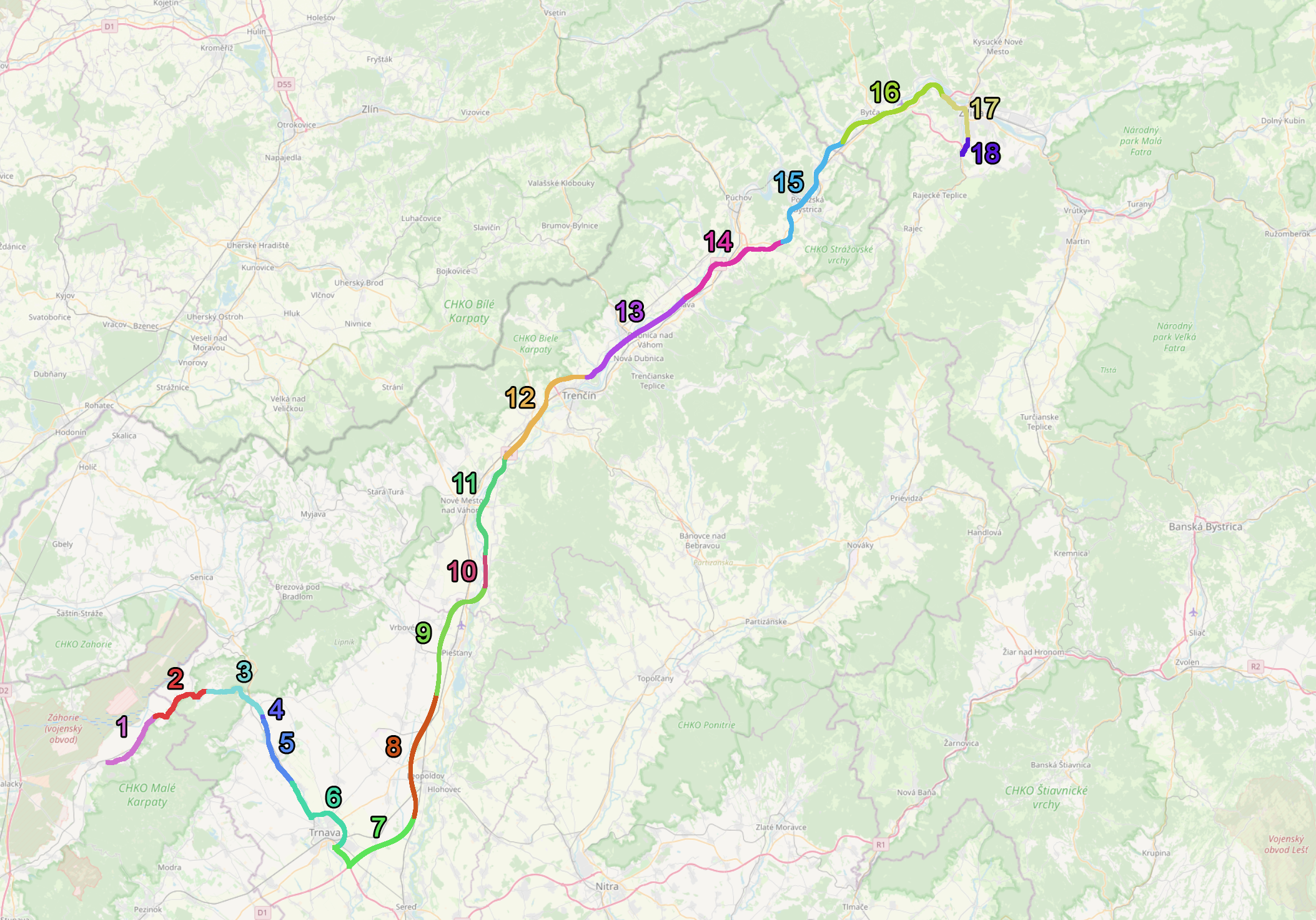

Supplement: Supplementary file 1 [file sensors-21-02881-s001.zip › Figure S3 - Division of the monitored route into sections.png]
